# Personalized Clustering for Emotion Recognition Improvement

**DOI:** 10.3390/s24248110

**Published:** 2024-12-19

**Authors:** Laura Gutiérrez-Martín, Celia López-Ongil, Jose M. Lanza-Gutiérrez, Jose A. Miranda Calero

**Affiliations:** 1 Departamento de Tecnología Electrónica, Universidad Carlos III de Madrid, Avenida de la Universidad, 30, 28911 Leganés, Spain; 2 Instituto de Estudios de Género, Universidad Carlos III de Madrid, Calle Madrid, 126, 28903 Getafe, Spain; 3 Departamento de Ciencias de la Computación, Escuela Politécnica Superior, Universidad de Alcalá, 28805 Alcalá de Henares, Spain; jm.lanza@uah.es; 4 Embedded Systems Laboratory, Ećole Polytechnique Fédérale de Lausanne (EPFL), 1024 Vaud, Switzerland; jose.mirandacalero@epfl.ch

**Keywords:** clustering, semi-personalized AI, user typology, unlabeled data, exportable methodology, affective computing

## Abstract

Emotion recognition through artificial intelligence and smart sensing of physical and physiological signals (affective computing) is achieving very interesting results in terms of accuracy, inference times, and user-independent models. In this sense, there are applications related to the safety and well-being of people (sexual assaults, gender-based violence, children and elderly abuse, mental health, etc.) that require even more improvements. Emotion detection should be done with fast, discrete, and non-luxurious systems working in real time and real life (wearable devices, wireless communications, battery-powered). Furthermore, emotional reactions to violence are not equal in all people. Then, large general models cannot be applied to a multi-user system for people protection, and health and social workers and law enforcement agents would welcome customized and lightweight AI models. These semi-personalized models will be applicable to clusters of subjects sharing similarities in their emotional reactions to external stimuli. This customization requires several steps: creating clusters of subjects with similar behaviors, creating AI models for every cluster, continually updating these models with new data, and enrolling new subjects in clusters when required. An initial approach for clustering labeled data compiled (physiological data, together with emotional labels) is presented in this work, as well as the method to ensure the enrollment of new users with unlabeled data once the AI models are generated. The idea is that this complete methodology can be exportable to any other expert systems where unlabeled data are added during in-field operation and different profiles exist in terms of data. Experimental results demonstrate an improvement of 5% in accuracy and 4% in F1 score with respect to our baseline general model, along with a 32% to 58% reduction in variability, respectively.

## 1. Introduction

Affective computing is an interdisciplinary field focused on observing, analyzing, interpreting, recognizing, and expressing human emotions. In recent years, it has gained significant attention [1]. This technology utilizes various types of sensor data to create systems capable of perceiving and interpreting human reactions to external stimuli, allowing for tailored responses. Recent developments in affective computing include emotional speech processing, facial expression recognition, body gesture analysis, multimodal systems, and the understanding and generation of emotions.

More specifically, significant advancements have been made in emotion detection, resulting in improved accuracy, reduced inference times, and user-independent models [2]. The use of huge and powerful processing units (GPUs, multicore servers, etc.) allows the implementation of complex models running in the cloud, with servers working 24 days/7 h and consuming huge amounts of energy. Alternatively, some commercial solutions allow local processing by applying specific microprocessors in the edge instead of cloud computing, usually focusing on non-critical applications [3,4,5]. However, there are critical applications running on the edge that require real-time operations with low power consumption, cybersecurity, and data protection mechanisms. For example, the Bindi system is an effective Internet of Things solution to combat gender-based violence [6]. In this scenario, lightweight AI models are required to operate on the edge and share as little information as possible outside this layer. This AI modeling simplification can be addressed by focusing on the clustering-wise personalization of models, meaning simpler models with better generalization capacities.

The research performed on emotion detection in people through the analysis of physiological and physical data has shown that there are very different emotional reactions in persons, depending on intrinsic and extrinsic factors [7]. Therefore, building a subject-independent model for emotion recognition is not the best option, as it generalizes the responses of all users. However, generating subject-dependent models makes the enrollment process and the application deployment almost unaffordable in most cases due to the large amount of data required for every model. In this dilemma, clustering persons according to similarities in emotional responses would avoid subject-dependent models and facilitate new user enrollment.

On this basis, this work introduces a personalized clustering approach to establish an effective, personalized, and precise emotion detection system oriented to edge-based applications. The key contributions include the following:

- A novel clustering methodology that leverages physiological response patterns to develop semi-customized models tailored to individual typologies, improving generalization and resilience in diverse user populations.
- An innovative method for integrating new, unlabeled users without the need for complete system retraining. By incorporating these users into pre-existing clusters and aligning them with trained models, the system supports seamless user enrollment without requiring labeled data, thus improving scalability and operational efficiency in real-world applications.
- A validation with an actual dataset containing measures and labels from 47 users has been done. Improvements in quality metrics have been observed, especially regarding standard deviation, with regard to similar works in the literature.

The paper is organized as follows. Section 2 states the assumptions and basic elements of the emotion detection system and the current issues faced by researchers. Section 3 presents the proposed methodology, and Section 4 details the tools used in the research presented in this paper and the experimental procedures followed to test both methodologies. Section 5 shows the experimental results. Finally, in Section 6, the authors discuss the results and conclude.

## 2. Background

The detection of emotions in humans using technological solutions has interested researchers in the last decades. The range of applications is widening every year. Starting with positive emotions identification, such as in marketing techniques to motivate potential shoppers or in entertainment and leisure activities to improve experiences, and going through negative emotions detection, in cases of mental health, surveillance, or violence prevention, there have been several contributions in scientific research and commercial devices, especially in the 21st century [1].

There is no global agreement on the range of emotions to be detected, neither their number nor their representation. However, the most accepted classifications are for discrete emotions, Ekman’s proposal [8], with 12 main emotions (joy, sadness, surprise, contempt, hope, fear, attraction, disgust, tenderness, anger, calm, and tedium), and for dimensional modeling, Mehrabian’s proposal [9], with the PAD space (Pleasure, Arousal, Dominance).

Automatic emotion recognition is based on AI algorithms that analyze one or multiple measurements from the person under analysis and classify the subject’s emotional reaction. This classification is done without the user’s participation, although subsequent confirmation is welcome to readjust the AI model. The key issue in affective computing is the type of variables measured to infer the emotional state. Those variables can be physiological signals (skin temperature (SKT), galvanic skin response (GSR), heart rate (HR), Electromyogram (EMG), Electrocardiogram (ECG), Electroencephalogram (EEG), etc.), instant reactions in the amygdala and sympathetic nervous system (SNS), and provoking the fight-or-flight natural reaction.

There has been intensive research about the best collection of variables to recognize emotional reactions with AI. The scientists have finally agreed that multimodal systems will provide better results than uni-modal ones. However, the number of sensors or measuring devices should be small so that the system can be worn easily while keeping low AI complexity to allow quick emotion detection [10].

An emotion recognition system is comprised of three primary components: sensors, data, and algorithms. The initial step involves identifying the specific variables to be measured, which will determine both the number and type of sensors to be utilized within the system. The subsequent step entails the collection of pertinent data from these sensors—specifically, information gathered from individuals during emotional experiences, accompanied by the appropriate emotional labels. These data will serve to train the artificial intelligence algorithm, enabling it to develop a model capable of accurately classifying emotions when presented with new data from either the same or different users. The performance of the system is assessed through various metrics that evaluate the quality of emotion classification. Depending on the data used for the training and testing and the algorithms employed (KNN, SVM, NN, etc.), the system quality can vary from 60% to 80% of correct answers. The bias avoidance in the data collection, a wide range of ages, both sexes, different and effective stimuli to provoke emotions, and a good labeling system, among other factors, will produce good datasets to train and test the algorithms.

Furthermore, more problems appear when the system is deployed and applied to new users. The real-life conditions, external situations, multiple stimuli, different sensors, etc., will produce different measurements and, possibly, different emotional reactions, which will confuse the inference model. The generation of a subject-independent model appears in the literature as a complex idea that may not be feasible for all users. Still, subject-dependent models are costly in terms of time and resources (specific data are required for every user). Clustering users’ data or other variables seems a good solution [11].

There are various works in the literature dealing with the use of clusters for AI algorithms focused on human reactions. In [12], Sarwar et al. propose an efficient method to improve the marketing techniques in e-commerce. Instead of clustering users by similarities and recommending items bought by users in the same group, the solution proposed clusters items bought together and recommends users with items in the same cluster, according to the shopping cart. The addition of new elements in the clusters requires analyzing the purchasing history offline, while online recommendations for users take very few seconds. In this work, the data managed included users, items, and relations.

In [13], the authors propose using clusters to improve the learning process in students based on the assessment questions with lower success rates. These clusters classify questions and, therefore, contents that allow the application of reinforcement schemes in a learning management system. The data processed were questions in the examination and assessments obtained. The clusters were created with respect to question difficulty and item discrimination, leveraging the number of students. However, the enrollment of new users (students) implied recalculating the clusters.

If we focus on emotion detection, clustering has grown in recent years. First, analyzing the utility of physiological signals in this detection, ref. [14] propose the use of typologies to determine the best variables for recognizing emotional states (with a coarse classification: positive–negative or neutral–non neutral). Nevertheless, the addition of new measurements is not considered. The data managed were physiological signals and emotion labels (target and self-reported).

Also, in [15], they apply clustering in emotion recognition through semantic analysis in conversations. The features extracted from the data collected are grouped into clusters located in a 3D PAD space, which are generated to reduce the high dimensionality. Although not every emotion is well detected, this proposal allows better and simpler AI models.

More recently, ref. [16] details the use of AI with KNN clustering for physiological variables (ECG and SKT) to generate a subject-independent model to classify the emotional state of intruders in secured environments. The addition of a new user in the system does not modify the original inference model. This scheme is proposed frequently in the literature. Ref. [17] classify emotions in students learning a foreign language with a multimodal approach (facial expression, audio, texts, and biological signals). With so many variables and features extracted, clusterization can be used to identify the most representative variables and generate a subject-independent inference model. The addition of a new user does not modify the original AI model.

Some authors treat the differences in emotion elicitation by considering the same external stimuli, not only labeling the emotion felt but also regarding the variation in their physiological variables. According to [7], previous experiences, post-traumatic stress disorders, personal traits, and current circumstances condition significantly affect the emotional reaction and its length of time. These differences imply two main difficulties in classifying emotions with AI algorithms. First, the dataset used for training and testing the algorithm should include those differences in emotional reactions, but if yes, a unique AI model will be very inaccurate. Previous clustering to identify similar emotional reactions would reduce this problem in a midterm between subject-dependent and subject-independent AI models. Secondly, a new user enrollment to the AI model should imply a previous analysis of which cluster the subject belongs to.

In [18], differences in emotional labeling are tackled. First, a subject-independent AI model is generated to classify the emotions felt by users. Secondly, those data with incorrect emotional classification are clustered and re-labeled, considering data with good classification results. Data used in this research are EEG signals from 11 and 32 channels. The addition of a new user is not considered.

Regarding personalized model generation, considering data for multiple users, we find two relevant works. First, the authors in [19] generated only one AI emotion detection algorithm, although the addition of new users is also considered. Physiological signals used are ECG, SKT, and GSR. Automatic feature calibration is generated for new users within the emotion detection system prior to the classification model. Therefore, different sensors and/or conditions are overcome. Users’ data are clustered with an unsupervised learning method, generating different centroids, which are used for comparison with the centroids of new users in the future. The best-correlated centroid will be used to calibrate the new data received and recalculate the CNN, with the objective of a subject-independent model.

Secondly, in [11], the real implementation of an emotion detection system is considered. The physiological data used are EEG, ECT, EMT, and GSR. Therefore, different users recruited in different moments, with different sensors and conditions, could be included in the emotion detection systems thanks to users’ clustering and one AI model per cluster. Each cluster would produce a different number and type of features to generate its model. The experiments performed for generating data from every user follow a similar scheme: baseline (no emotion), task (stress emotion), and recovery (no emotion). The data used for cluster generation are those from the “baseline” stage. Therefore, few differences are found among users, and only two clusters are generated. The rest of the data are used to generate the AI models. This, together with the amount of data compiled (41 users, 11-11-19 30 s windows, 50% overlapped), makes it necessary to reconsider the approach.

To our knowledge, there is not an efficient methodology to include new users in emotion detection systems with a subject-dependent model based on clusters. Even so, such a methodology is not successfully defined for any AI model dealing with different sources of data. Two methodologies are proposed in this work: first, a methodology (M1) to cluster labeled data from users, employed to build the AI model (generating a semi-customized model for every cluster), and a methodology (M2) to ensure the enrollment of new users with unlabeled data. Furthermore, these methodologies are conceived to work on the edge, avoiding cloud computing, which would provoke insecurity in data protection, higher power consumption, and longer inference times. The solution presented in this paper fits with personalization, accuracy, and scalability.

## 3. Proposed Clustering Methodology

This paper proposes two methodologies designed and implemented to explore the potential of identifying typologies of input data and how these groupings can lead to personalized and improved AI models. These two methodologies are defined to be applied in sequence: first (M1) to identify clusters in the input data and generate the semi-customized AI models, and second (M2) to enroll new users (user, volunteer, and participant are used interchangeably in this paper) in the AI model. The idea is that this complete methodology can be exportable to any other expert systems where unlabeled data are added during in-field operation and different profiles exist in terms of data.

In the first approach, M1, the volunteers are grouped into distinct categories or clusters based on statistical variables corresponding to the labeled observations. This technique requires acquiring multiple labeled observations from all target classes for the same participant.

The second approach, M2, is an extended version of M1 that brings the system as close as possible to a real use case. This scenario starts with clusters already generated by applying M1. Then, new unlabeled volunteers are automatically assigned to specific clusters, taking advantage of the customized model’s benefits.

For both cases, an optimal cluster search algorithm is required to identify the best number of groups within the data. The detailed information of the characteristics and configuration is described in the following subsection.

The methodologies are applied and validated onto an emotion-detection system that receives two types of input data: users’ emotion labels and physiological signals measured.

### 3.1. Optimal Cluster Search Algorithm

Clustering is a fundamental technique in the field of data mining and serves as a crucial tool for identifying patterns within unlabeled data based on shared characteristics. The existing literature features several comprehensive reviews [20] that examine a variety of clustering methodologies and metrics. In this regard, the authors have selected the hierarchical algorithm, which is a well-established and adaptable strategy. This algorithm is particularly effective in revealing nested relationships within datasets.

On this basis, the search algorithm, guided by the Dunn index, identifies the optimal number of clusters (the exploration space ranging from two to ten) by employing the hierarchical clustering method using the Euclidean distance metric and ward linkage criteria. The Dunn index is a metric for evaluating clustering quality. Its primary objective is to maximize the separation between clusters while simultaneously minimizing the dispersion within each cluster. This attribute renders the method particularly advantageous for the validation of clustering results and the selection of optimal partitions, especially in contexts where the number of clusters or the distribution of the data has not been predetermined. It is derived from the ratio between the minimum inter-cluster distance and the maximum intra-cluster distance [21].

An experimentation-based prerequisite condition is imposed to ensure that each resulting cluster incorporates a minimum representative size of 15% of the total dataset to facilitate the AI model having enough data to generate a robust model and prevent the formation of excessively small clusters that may only represent a few outliers. If a cluster fails to meet this criterion, it is merged with the nearest cluster. Accordingly, once the clusterization is done, the size requirement is checked; if the clusters fulfill the condition, the final number of clusters will match the optimal number found by the search algorithm. Otherwise, the clusters below the minimum size will be absorbed by the one closest to them, and in this case, the final number of clusters will be smaller than the optimal one previously found.

### 3.2. User Profile Clustering Based on Labeled Data (M1)

This first technique, M1, aims to identify user typologies of users for the AI model. The underlying concept is to detect analogous responses to enhance data similarity, thereby facilitating the model in discerning between different classes. This approach enables a semi-personalized yet broader model compared with a subject-dependent one.

**Figure 1:**
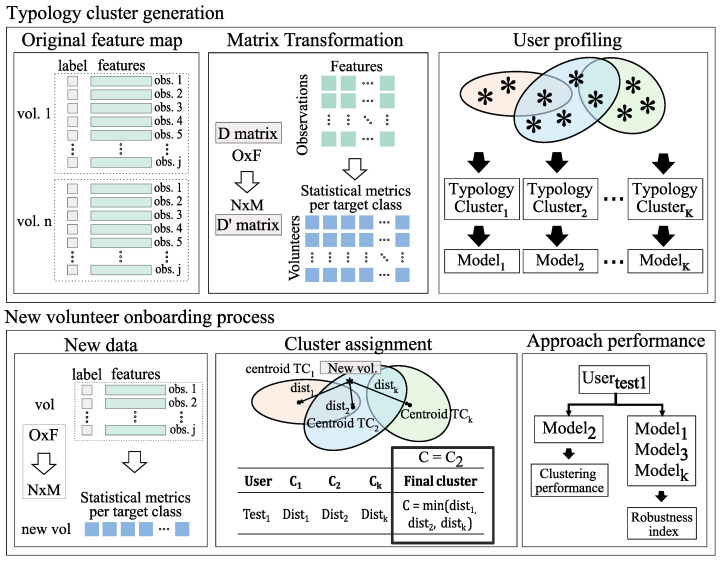
User profile clustering based on labeled observations training and testing scheme.

As the first line of Figure 1 depicts, the process starts generating the feature map *D*, an O×F matrix containing the *F* features for the *O* observations used to generate the AI models. Then, a $R^{O\times F}\to R^{N\times M}$ transformation of the feature map *D* is required, getting $D^{\prime }$, where *N* represents the number of volunteers and *M* includes the mean and standard deviation of all the features for each of the target classes.

Next, $D^{\prime }$ feeds the optimal cluster search algorithm exposed before, getting *K* optimal clusters and their corresponding centroids $C_{k}\in R^{M}$, k∈{1,...,K}. Finally, a specific AI model will be trained for each of the clusters, using the previously assigned volunteers during the clustering.

The second line of the scheme presents the assignment of a new volunteer within the system; an initial computation of the transformed feature matrix is needed, and then, by employing the minimum distance technique, the user is designated to one of the existing TCs. These cluster assignments are validated following the approach performance block shown in the figure. This validation is detailed in Section 5.1.

### 3.3. Unlabeled Observation Clustering Assignment (M2)

This approach aims to extend the proposed user clustering methodology in M1 to enable the assignment of any unlabeled observation to the closest profile, thereby creating a more complex but realistic technique. With this intention, the clusterization done by M1 is considered the starting point of the system. To aid understanding and maintain consistency, clusters found in M1 are referred to as typology clusters (TCs), and the new clusters computed in this method are referred to as Internal Clusters (IC).

The primary challenge in assigning new observations, $O\in R^{F}$, to an existing TC lies in the dimensional disparity between these two points (the centroids and the observation). To mitigate this issue, the TCs centroids ($C_{tc}\in R^{M}$) must be redefined to align with the dimensions corresponding to the features associated with each data instance, $R^{F}$. This is going to be possible through the ICs.

To do this, all the training observations linked to each of these TCs are used to compute from four to six ICs that represent the behavior within each TC in terms of observations, ensuring that their centroids $C_{ic}\in R^{F}$. These ICs are found using the algorithm to select the optimal number of groups.

**Figure 2:**
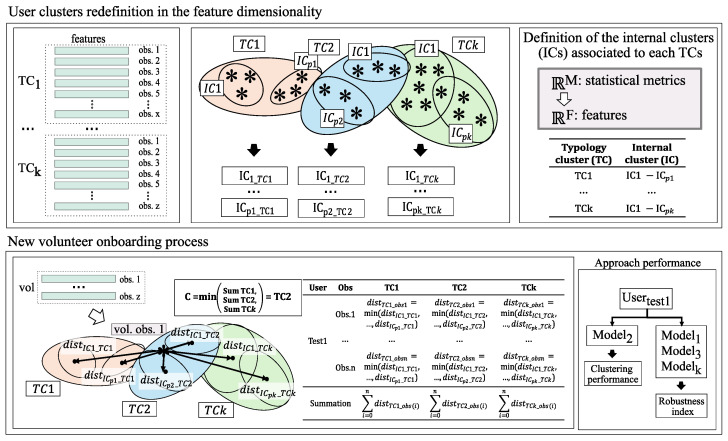
Unlabeled observation clustering assignment scheme.

Finally, when integrating a new volunteer into a specific TC, the cluster is selected based on the lowest summation of distances of the available observations to the closest IC centroids of each TC. The data pipeline is presented in Figure 2.

## 4. Materials and Methods

The following section provides detailed information concerning the experimental methodology, which is divided into three phases, and the tools necessary to implement the proposed methodology. The material includes the dataset, the machine learning pipeline, and late fusion strategies to test the influence of the clustering strategy over the fear classifier.

The complete pipeline and model implementation are developed using Python v3.8.10, leveraging its Scikit-learn and SciPy libraries.

### 4.1. Dataset

This research utilized the first release of the women and emotion multimodal affective computing (WEMAC) data [22,23], a multimodal dataset integrated within the UC3M4Safety repository. The UC3M4Safety research team compiled and disclosed this dataset between 2020 and 2023. Its principal aim is to comprehend and formulate models delineating the relationship between physiological signals and fear activation [24].

This first set contains data from 47 women volunteers exposed to 14 validated audiovisual stimuli through a virtual reality environment. After each visualization, an interactive screen allows the participants to self-report the emotional labels.

At the same time, their physiological information (galvanic skin response (GSR), skin temperature (SKT), and blood volume pulse (BVP) signals) is collected utilizing the BioSignalsPlux research toolkit [25]. Specifically, the raw and filtered signals are the ones released and publicly available in the e-cienciaDatos portal [26], a research data repository held by ‘*Consorcio Madroño*’ (composed of 250 universities in the Madrid, Spain region and is a member of the Harvard Dataverse Network, accessible for publishers).

A complete data descriptor of the experimental procedure, ethical statements, participants, stimuli, measures, data processing and cleansing, libraries and software used, and apparatus is detailed in [23].

The pre-processing steps applied to obtain the filtered signals are as follows: On one hand, two filters are used to address noise issues in the BVP signal. A direct-form low-pass FIR filter with 6 dB at 3.5 Hz filters out high-frequency noise. A forward–backward low-pass Butterworth IIR filter removes the low-frequency drift effect in the signal. On the other hand, GSR and SKT signals are filtered with a 2 Hz cut-off frequency and processed with moving average and median filters. The moving average uses a 1-second window to reduce noise, while the moving median uses a 0.5-second window to deal with rapid transients [6,27,28].

The resulting denoised signals are at sampling frequencies of 100 Hz. These are selected for the following steps.

Finally, of the 47 volunteers, three were discarded from experimental procedures due to virtual reality sickness [29,30] and some signal anomalies caused by technical problems.

### 4.2. Machine Learning Pipeline

The designed model to test the impact of the methodology on fear classification performance is a binary supervised KNN machine-learning classifier. It is an adapted version of the Scikit-learn model, incorporating the misclassification cost as a new hyperparameter.

A stepwise Bayesian optimizer with five cross-validation folds is employed to determine the optimal hyperparameters following the F1 score as the scoring metric. Among the parameters to be tuned are the misclassification cost or positive class weight (ranging from 0.5 to 2.5), the number of neighbors (ranging from 20 to 100), and the distance metric (Euclidean and Manhattan).

Additionally, a feature selection step is incorporated during the training stage; one filter (minimal Redundancy and Maximum Relevance, mRMR), one wrapper (sequential forward selection, SFS), and one hybrid (mRMR-boruta) method are tested [31,32].

The final model performance is evaluated using the accuracy and F1 score and their standard deviations. Its implementation is based on previously tested versions using the same dataset. Therefore, its performance is preliminarily validated by comparing it with results from prior studies conducted in Matlab®. The selection of the KNN model was the result of a comprehensive analysis between the KNN, SVM, and ensemble models, and careful consideration was given to the implications of deploying a deep learning model, with a size ranging from 300 kB to 1 MB, on an extremely low-power device characterized by limited Flash and RAM capacity. Such constraints may potentially interfere with the execution of other critical tasks [10].

### 4.3. Experimental Procedure Pipeline

The complete pipeline for testing the methodology is divided into three phases: (1) data preparation, (2) cluster generation, and (3) model training and performance evaluation.

**Figure 3:**
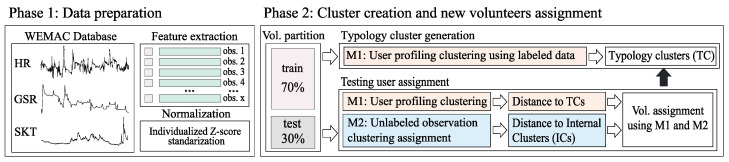
Scheme of phase 1 and 2 of the experimental procedure for evaluating the impact of the methodologies on fear detection.

In Figure 3, a schematic of the two first blocks is presented. In the first stage, an initial pre-processing step for feature extraction is shown. It uses 20-second windows with a 10-second overlap for computing 57 features. It extracts 31 features for BVP, including four features in the time domain, 12 features in the frequency domain, and 15 non-linear features. Similarly, it extracts 20 features for GSR, which includes nine features in the time domain, three in the frequency domain, and eight nonlinear. Finally, six features for SKT are extracted, including four features in the time domain and two in the frequency domain. Note that all the features considered are a compilation of the most relevant and successful ones based on well-established physiological expert knowledge related to emotions [33,34,35]. An extensive description of the features is provided in [27,28,36].

Then, a normalization step is applied to standardize the features using an individualized Z-score normalization for each volunteer.

Upon completion of data preparation, in phase 2, a random 70–30% partition of the volunteer dataset is created to facilitate testing of the two proposed methodologies. The 70% subset is utilized to generate typology clusters (TCs) using method M1, while the remaining 30% is assigned to one of these TCs using both methodologies.

**Figure 4:**
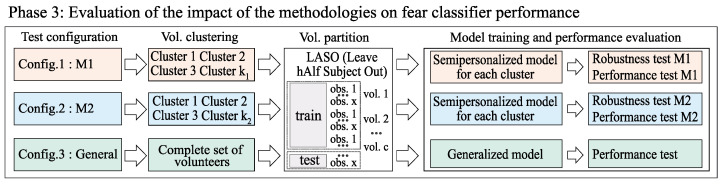
Scheme of phase 3 of the experimental procedure for evaluating the impact of the methodologies on fear detection.

Finally, once all the volunteers are assigned to one of the generated clusters, the testing procedure is designed. In the third block, Figure 4, three different configurations for evaluating the performance are set. In Config.1 and Config.2, each of the clusters found using M1 and M2 (Phase 2) are used to train and evaluate the semi-personalized models. On the other hand, in Config.3, the complete set of volunteers is included to create the general model that is considered as the baseline.

For all the configurations, a LASO (Leave hAlf Subject Out) strategy is proposed for training, validation, and testing. This strategy involves reserving half of the data of a single participant as the testing partition. The machine learning model used and the detailed pipeline followed to train and evaluate its performance are previously described in Section 4.2.

### 4.4. Late Fusion

Multimodal data fusion techniques are a common practice to exploit and combine complementary data from different modalities [37,38]. When dealing with emotion recognition, some comprehensive reviews can be found in [39,40].

In this work, the idea is to use these late combination techniques to merge the customized model for each cluster and the generalized one and study how the contribution of both models can help to improve the robustness of the results of the non-well-defined typologies.

As the KNN model infers a binary label, and both models are fed with the same data type, the merge output is directly computed using the predictions $p_{n}$ computed per observation or based on the entropy $h_{n}$ for the *n*-th time period as a metric to represent how confident the label predicted is. The entropy is calculated as

(1) $$h_{n}=-\left[p_{n}\cdot \log\left(p_{n}\right)+\left(1-p_{n}\right)\cdot \log\left(1-p_{n}\right)\right]$$

On this basis, four late fusion strategies are studied to generate a merge response. They are based on the literature [41] and are proposed as a trade-off between low computational complexity and robustness, considering the confidence of the personalized and general system in the predictions.

- Type 1—Contribution index: the system’s output is the merge of both labels based on the percentage contribution of each model, where 0 contribution index is the same as only using the semi-personalized model, and 1 implies only using the general model.
- Type 2—Lowest entropy: the system’s response corresponds to the binary label determined by the model with the lowest entropy. The entropy value is calculated based on the fear probability inferred for each video.
- Type 3—Inverse entropy weighted combination: the fused label is computed as the weighted sum of probabilities of fear $p_{n}^{f}$, where the weight ranges from 0 to 1 and comes from the inverse entropy $w_{n}$, as given by
  (2) $$p_{n}^{f}=\sum w_{n}\cdot p_{n},\mathrm{where}\;w_{n}=\frac{1/h_{n}}{\sum 1/h_{n}}$$
- Type 4—Logical OR: the final response is the logical OR computation over the binary labels for each model.

When comparing the four techniques inside, it is important to point out that Type 1 and Type 4 compute the final prediction without checking the confidence or comparing the quality of the models, which could lead to more errors. However, the main difference between those is that Type 1 combines the output of both models, which can add robustness. In Type 2, the strategy trusts the most confident model (lowest entropy) and forgets about the rest of the information. Finally, Type 3 not only gives importance to the confidence of the model but also takes into account the probability of each of the models and uses both models to generate a final prediction.

## 5. Results

This section details the experiments conducted to validate and test the proposed methodology and how it proceeds and influences the performance metrics of the KNN classifier.

The analysis is organized into three main blocks. The first section, Block A, offers a detailed examination of the configurations proposed in Section 4. The performance metrics are divided into three validation indices: (1) Robustness test, where the model is tested with the volunteers that do not belong to that specific cluster; (2) Performance test, where the clustering model is tested with its assigned and unseen volunteers; (3) Volunteers assignment comparison test between methods.

The second section, Block B, highlights the potential improvements achieved through the use of fusion techniques. Finally, the last section, Block C, presents a comparative study of the results, which includes groupings by typology, the general model, and an overview of the current state of the art.

### 5.1. Block A: Methodologies Performance

In Block A, the experimental procedure presented in Section 4 is followed.

In the context of our designated use case, as previously detailed, methodology M1 is implemented by taking into account the emotional and physiological responses of 70% of the participants after the data preparation phase. A feature map, *D*, is constructed utilizing the 57 extracted features, along with its corresponding matrix transformation. Consequently, $D^{\prime }$ comprises a single row for each participant, resulting in a total of 228 columns derived from the multiplication of the 57 features by two target classes (fear and non-fear) and two statistical metrics (mean and standard deviation).

As a result, the optimal number of TCs found by the search algorithm is four. As previously mentioned, based on distance, the rest of the volunteers (testing partition) are assigned to one of these centroids using their transformed feature matrix.

After that, with the intention of applying the user assignment proposed by M2, first, it is necessary to mitigate the dimensional disparity between these TCs centroids and the observations by means of the ICs. After that, using the minimum distance between observation and IC centroids, the volunteers are designated to one TC.

Once all volunteers are assigned to one of the TCs following both the M1 and M2 approaches, and with the clustering information, one general model and four different semi-personalized KNN models for each proposed technique are trained. Performance metrics are computed through a LASO strategy.

The validation results are displayed in Table 1. The values shown by cluster 1 and cluster 2 are particularly noteworthy, almost reaching and even surpassing the 70% threshold in the performance metrics, which exhibit significant improvement compared with the baseline. Cluster 3 is the next with the best results, although it stays between 65 and 68%. Cluster 4 shows poor values that hardly reach 60 for M1. In M2, this same group achieves results akin to those found in cluster 3.

In the final section of the table, the overall average metrics for the baseline model, as well as the weighted average metrics $m_{c}$ for the personalized models (Equation (3)), are calculated. The weighted indices are determined by considering the number of volunteers involved in each cluster, $n_{c}$. The results show a general improvement for both of the methodologies.

(3) $$m_{average}=\frac{n_{c1}\cdot m_{c1}+n_{c2}\cdot m_{c2}+n_{c3}\cdot m_{c3}+n_{c4}\cdot m_{c4}}{n_{c1}+n_{c2}+n_{c3}+n_{c4}}$$

The second validation index, robustness, tries to prove the stability of clustering partitions. For this, the performance of personalized models using the volunteers assigned to the other cluster is computed to demonstrate that the values are clearly below the clustering model one. In other words, the volunteers of the rest of the clusters worsen the numbers because they belong to a different typology. In all cases, a difference in the performance of at least 5% is observed, except for the accuracy metrics of cluster 4, which presents almost the same levels for both indices in M1. This shows that the typology represented in the fourth cluster works similarly with the volunteers assigned to this cluster and with the rest. However, when using the M2 assignments, these figures improve and are similar to the rest of the clusters.

Besides analyzing the performance metrics of accuracy and F1 score, and with the intention of verifying the distribution of the testing volunteers generated by both methods, a comparison between the M1 and M2 assignments to the same volunteer is made. A total of 84% of the total number of volunteers are assigned to the same cluster using both methods, which can be translated to similar results for both approaches.

The combination of all the previous results leads us to be able to say two things. The first is that both methods are equivalent in volunteer assignments; therefore, the use of the M2 has been validated despite its complexity. The second is that although some of the values for specific clusters, such as clusters 1 or 2, show clear improvements with respect to the baseline, especially in M1, M2 shows more stable figures among all groups. Considering these implications, only the results from methodology M2, which is the closest to a real case, will be considered.

### 5.2. Block B: Late Fusion Techniques

In Block B, different fusion strategies, previously defined in Section 4.4, are incorporated to test the effects of the weighted merge of both models (personalized and general) in the performance and study the possible implications of using personalized models when the specific cluster is not well defined.

The average performance metrics for each fusion case are detailed in Table 2. Several statements can be drawn from the results presented. Firstly, only the first type of merge (contribution index) shows a slight increase in metrics across all groups. In contrast, the fourth type of fusion technique (logical OR) results in a decline in performance, with metrics dropping between 2% and 13% across nearly all measurements. These figures align with the previously discussed theoretical weaknesses of this strategy.

In the analysis of types 2 (lowest entropy) and 3 (inverse entropy weighted combination), it is evident that clusters 1 and 3 have demonstrated notable improvements. Conversely, clusters 2 and 4 have experienced a decline in performance. This finding corresponds with the observation that clusters utilizing customized models tend to outperform those that use general models. Therefore, it can be concluded that clearly defined typology clusters lead to superior outcomes. In contrast, entropy analysis proves to be ineffective when the profiles are not clearly delineated.

**Figure 5:**
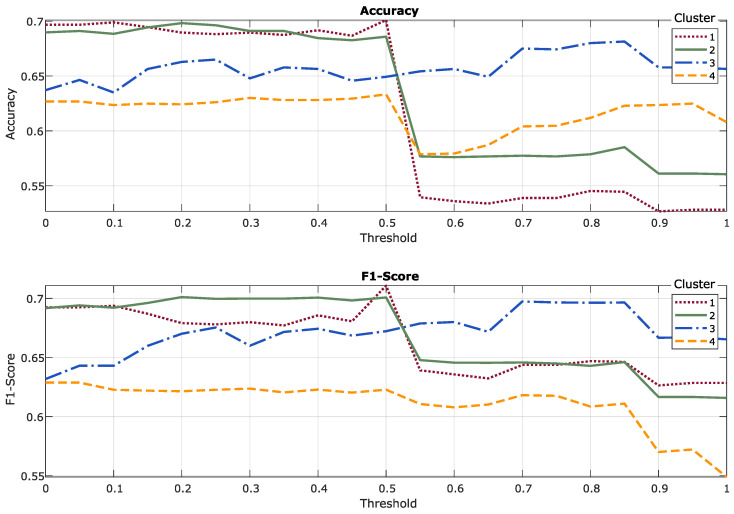
Average performance metrics (accuracy and F1 score) per typology cluster with a parameter sweep for the general model contribution threshold. 0: only personalized model; 1: only general model.

Additionally, a noteworthy finding is that cluster 3 demonstrates an approximate 5% improvement across all fusion techniques. This observation, illustrated in Figure 5, along with the selection of the optimal threshold in the first fusion technique, indicates that incorporating the generalized model provides additional insights. This enhancement is particularly significant, given that the typology of volunteers is still not well-defined, suggesting that the generalized approach positively impacts the overall metrics.

The graph includes the metrics for the four clusters in the Type 1 late fusion technique and illustrates how the metrics behave as the weight of the general model in the output label of the first fusion technique increases. Clusters 1, 2, and 4 experience a sharp decline in accuracy values once the threshold of 0.5 is surpassed. In contrast, cluster 3 exhibits a positive trend, achieving better results with a greater contribution from the general model. The F1 score graph displays similar trends, though the changes are somewhat more gradual.

### 5.3. Block C: Comparative Study

This section examines the mean results of the proposed techniques as well as the late fusion subsequently executed. Finally, it presents a comparative analysis of our results and the state-of-the-art works.

In Table 3, our methodology and similar works, their configuration, and finally, their accuracy and F1 score metrics are presented.

Among the results shown, it is first necessary to highlight the improvement obtained by the cluster methodology and, particularly, the type 1 fusion technique, which obtains values 11.5% and 11% higher than our baseline in accuracy and F1 score, respectively, and 32.6% and 58.8% less standard deviation. This last approach allows new volunteers to be integrated without the need for labeled data. By combining personalized and general models, the system is able to effectively tackle its various stages from a realistic perspective. In other words, the characteristics of cluster definitions can change over time based on the amount of integrated data. The quality of these clusters will enable this technique to adapt dynamically, determining whether it is more advantageous to use customized models or to depend on general information.

Regarding the state of the art, if compared with Bindi [6], the semi-personalized models show a slight improvement, an increase of 4.8% and 3.2% in accuracy and F1 score metrics, respectively, but a clear reduction of 70.6% and 79.1% in standard deviation. On the other hand, the results obtained by the authors in [42] are 10% above our values. However, despite the advantages in terms of performance, the model implemented in this work, EfficientNet, is not feasible in a wearable device with low power consumption, data protection, and short inference times due to its architectural complexity, extended training time, high memory usage, and specific hardware requirements. In fact, the presented results correspond to metrics computed in the GPU, and when the authors try to implement this same model in an on-the-edge device, they obtain 5% lower values and a mean power consumption of 2.10 W, a figure far away from a real feasible wearable system.

It is important to state the significant reduction in the standard deviation the methodologies M1 and M2 are providing; as previously mentioned, reductions of 32% and 58.8% are found for accuracy and F1 score, respectively, with respect to our baseline, but even a 70% and 79% are reached compared with the state-of-the-art Bindi model.

## 6. Conclusions and Future Work

This work proposes a novel clustering technique for semi-personalizing a general AI model and tackling the problem caused by the different physiological reactions to the same target emotion. This approach combines the initial utilization of user clustering based on labeled data to decide the existing typologies within the data with the possibility of assigning new volunteers to their appropriate model later (with unlabeled data).

The experimental results, including robustness numbers and the results of semi-personalized and fusion techniques, demonstrate slight improvements in final performance values and a clear reduction in variation, indicating better generalization of the problem. Improvements of 5 to 11% in accuracy and of 3.2 to 11% in F1 score in mean and from 32 to 71% in accuracy and from 58 to 79% in the standard deviation are achieved with M2 compared with baseline and Bindi [6], respectively. These quantitative improvements signify not only enhanced overall accuracy but also a reduction in the incidence of false alarms. In the context of a real-world system, these advancements would result in decreased discomfort for users, thereby increasing their likelihood of utilizing the devices more frequently.

Comparing results with those provided by [42], which uses an EfficientNet AI model, authors consider a migration to this new implementation is desirable, although challenging to implement on the edge. However, as it is also explained in the previous section, it is important to recognize the drawbacks of using an overly complex network architecture. High levels of complexity can lead to increased memory usage, which may necessitate specialized hardware. This reliance on specific hardware can hinder the system’s implementation on edge devices.

For that reason, future work will explore using more complex deep learning models, such as convolutional neural networks and long short-term memory networks (LSTMs). These models have demonstrated notable effectiveness in identifying spatial correlations and temporal patterns, which are capabilities that KNN models lack and significantly restrict their effectiveness in addressing this type of problem. But also some optimizations for the assignment or clustering techniques. Nevertheless, it is essential to maintain a focus on the objective of implementing these techniques within extremely low-energy devices.

Special considerations and limitations of the proposed work must be considered. On the one hand, it is important to notice that fixing a specific hierarchical clustering with the Dunn index is a bias that we are adding to the methodology, and probably, a more extensive analysis of the effects of different strategies would strengthen the work. On the other hand, for testing the generalizability and robustness of the methodology, it would be beneficial to use more than one dataset, even with different physiological signals, as the complete configuration could be biased for this specific use case. Another important limitation of the study is the number of volunteers. There exists a possibility that the lack of data is preventing us from detecting other typologies that are currently agglomerated in the same cluster. For that reason, the authors are currently performing this same study with a more extended dataset, the second release of data from the same database used [23], and more focused on the deep learning paradigm. Even conducting a temporal study to emulate a real-world system, wherein new data are introduced at various chronological intervals, should be regarded as a critical future step of this work. In any case, the authors perceive typology clusters as dynamic entities that must evolve in accordance with the growing volume of data over time. This ongoing process may result in the creation of new typologies, the subdivision of existing ones, or even the elimination of some. Each group will exhibit varying degrees of clarity in its defining characteristics. This is where the fusion technique Type 1 comes into play. It allows for the integration of semi-personalized models with general models. If any of the clusters is not clearly defined, the general information can be utilized to classify fear more effectively.

## Figures and Tables

**Table 1:** Average performance metrics (accuracy and F1 score) over 20 cross-validation iterations for both methodologies and per typology cluster. Mean and standard deviation between brackets. The best results for each of the approaches are highlighted in bold.

Typology Cluster	Validation Test	Methodology M1		Methodology M2	
		Accuracy	F1 Score	Accuracy	F1 Score
C1	Robustness test	53.11 (7.23)	57.11 (6.45)	56.17 (7.23)	59.24 (6.61)
	Clustering $model_{1}$	79.21 (2.13)	73.09 (2.16)	69.67 (4.71)	69.24 (6.47)
C2	Robustness test	50.10 (6.94)	48.21 (10.05)	53.15 (8.08)	49.49 (10.69)
	Clustering $model_{2}$	68.97 (5.92)	67.17 (3.38)	68.15 (5.19)	68.01 (5.74)
C3	Robustness test	57.57 (7.02)	57.90 (7.37)	54.87 (11.22)	53.64 (13.14)
	Clustering $model_{3}$	65.71 (2.61)	66.18 (2.32)	63.09 (5.74)	63.58 (5.06)
C4	Robustness test	56.73 (7.93)	55.48 (11.91)	56.47 (6.31)	54.19 (11.95)
	Clustering $model_{4}$	58.86 (5.70)	61.88 (3.81)	62.68 (7.11)	62.87 (3.92)
All	Weighted personalized models	68.95 (4.23)	67.49 (2.79)	66.21 (5.59)	66.20 (5.39)
	General baseline model	60.75 (7.23)	61.93 (8.79)	60.75 (7.23)	61.93 (8.79)

**Table 2:** Average performance metrics (accuracy and F1 score) with and without late fusion techniques per typology cluster. Mean and standard deviation between brackets.

Typology Cluster	Performance Metrics	No Late Fusion	Type 1		Type 2	Type 3	Type 4
C1	Accuracy	69.67 (4.71)	th. 0.5	70.01 (2.61)	70.71 (7.06)	69.88 (6.21)	56.71 (7.02)
	F1 score	69.24 (6.47)		71.07 (2.84)	62.41 (6.10)	64.29 (5.69)	62.19 (4.61)
C2	Accuracy	68.15 (5.19)	th. 0.2	69.82 (5.50)	63.64 (4.52)	60.22 (4.99)	51.91 (6.84)
	F1 score	68.01 (5.74)		70.11 (3.02)	57.05 (6.43)	56.68 (7.42)	55.32 (5.23)
C3	Accuracy	63.09 (5.74)	th. 0.7	67.50 (3.56)	67.89 (5.28)	66.63 (5.46)	61.31 (5.64)
	F1 score	63.58 (5.06)		69.73 (4.95)	66.23 (5.31)	68.06 (5.82)	67.44 (5.79)
C4	Accuracy	62.68 (7.11)	th. 0.05	62.71 (8.30)	56.87 (8.23)	57.65 (7.29)	55.04 (6.34)
	F1 score	62.87 (3.92)		63.28 (3.81)	57.81 (5.61)	57.24 (6.35)	56.83 (6.43)

**Table 3:** Comparison of the proposed methodology’s performance metrics (accuracy and F1 score) with the related works. Mean and standard deviation between brackets.

Methodology	Model Configuration	Accuracy	F1 Score
Bindi [6]	KNN with SFS	64.63 (16.56)	66.67 (17.31)
Sun et al. [42]	EfficientNet with Recursive Feature Elimination (CNNs)	79.90 (4.16)	78.13 (6.52)
General baseline model— without clustering	KNN with mRMR	60.75 (7.23)	61.93 (8.79)
M1 clustering model: user profile clustering based on labeled data	KNN with mRMR	68.95 (4.23)	67.49 (2.79)
M2 clustering model: unlabeled observations clustering assignment	KNN with mRMR	66.21 (5.59)	66.20 (5.39)
Fusion Technique Type 1: M2	KNN with mRMR	67.74 (4.87)	68.78 (3.62)

## Data Availability

The use of the WEMAC dataset is licensed under a Creative Commons Attribution 4.0 International License (CC-BY-4.0). The data are hosted in the UC3M4Safety Repository in the ‘*Consorcio Madroño*’ online platform at https://edatos.consorciomadrono.es/dataverse/empatia, (accessed on 18 November 2024). The complete data descriptor and the instructions to decrypt the information are detailed in [23].
